# Subscription-Boxes in Out-Patient Departments

**Published:** 1886-11-06

**Authors:** H. Howgrave Graham

**Affiliations:** Hospital for Epilepsy and Paralysis, Regent's Park


### SUBSCRIPTION-BOXES IN OUT-PATIENT DEPARTMENTS.

Referring to the paragraph in your last issue on the smallness of the contributions placed in subscription-boxes in out-patient departments, one would certainly expect that in a large hospital the amount would far exceed £5. I find that at this comparatively small institution, where the "attendances" of out-patients scarcely exceed 8,000 per annum, we found as much as £8 1*s.* 9*d.* in the box in the waiting-room during the year 1885. As you point out, however, the amount received from the "paying" out-patients (also voluntary, but at a weekly rate fixed by the patients themselves) exceeded £271. The £8 1*s.* 9*d.*, no doubt, came from the pockets of the free patients. The palatial grandeur of some of our larger hospitals would no doubt have a deterrent effect on the out-patients.

H. Howgrave Graham.

Hospital for Epilepsy and Paralysis,
Regent's Park.

